# Severe Hypophosphatemia Induced by Hyperventilation: A Case Report

**DOI:** 10.7759/cureus.95048

**Published:** 2025-10-21

**Authors:** Jayakrishnan Jayakrishnan, Nikitha Narayanan, Thrasos Macriyiannis

**Affiliations:** 1 Acute Medicine, University Hospitals of Leicester NHS Trust, Leicester, GBR; 2 Geriatrics, University Hospitals of Leicester NHS Trust, Leicester, GBR; 3 Endocrinology and Diabetes, Leicester Royal Infirmary, Leicester, GBR

**Keywords:** acute medicine, anxiety, electrolyte imbalance, hyperventilation, hypophosphatemia, panic attack, respiratory alkalosis

## Abstract

Hypophosphatemia is a clinically significant but often underrecognized electrolyte abnormality in acute medicine, arising from decreased intake, increased losses, or intracellular redistribution. One uncommon cause is hyperventilation-induced respiratory alkalosis, which promotes intracellular phosphate shifting and enhanced glycolysis, leading to depletion of serum phosphate. Severe hypophosphatemia may result in neuromuscular, cardiovascular, and metabolic complications, necessitating prompt recognition and management. We report a case of a 38-year-old male patient referred to the Accident and Emergency (A&E) department by his general practitioner with severe hypophosphatemia (serum phosphate <0.20 mmol/L) and an elevated lactate level (2.4 mmol/L). He presented with shortness of breath, paresthesias of the upper and lower limbs, near-collapse episodes, hunger, sugar cravings over four months, and recurrent diarrheal episodes (six to eight per month). His history included Crohn’s disease, anxiety disorder, muscle cramps, and recent weight gain (~6 kg). On examination, he demonstrated hyperventilation, with arterial blood gas showing respiratory alkalosis (pH 7.535). Investigations revealed normal calcium (2.6 mmol/L; reference range 2.1-2.6mmol/L) and ionized calcium on venous blood gas (1.27 mmol/L; reference range 0.8-1.5 mmol/L), normal thyroid function (thyroid-stimulating hormone (TSH) 1.5 mU/L), and no evidence of malabsorption or renal phosphate wasting. Differential diagnoses, including malabsorption syndromes, vomiting, hyperparathyroidism, Fanconi syndrome, and refeeding syndrome, were excluded. The patient received intravenous phosphate replacement with serial electrolyte monitoring to assess response and prevent hypocalcemia, followed by transition to oral phosphate supplementation. Symptoms resolved completely, and the serum phosphate level normalized. Hyperventilation related to anxiety likely caused excessive CO_2_ loss, triggering respiratory alkalosis, which increased intracellular phosphate utilization and lactate production. Although often overlooked, severe hypophosphatemia can cause muscle weakness, rhabdomyolysis, hemolysis, arrhythmias, and altered mental status if untreated. This case underscores the importance of recognizing metabolic disturbances in patients presenting with panic-like hyperventilation and supports early electrolyte assessment to prevent serious complications.

## Introduction

Hypophosphatemia is an uncommon yet clinically significant electrolyte disturbance frequently encountered in acute medical settings. Phosphate plays a crucial role in cellular energy metabolism through adenosine triphosphate (ATP) generation, maintenance of cell membrane integrity via phospholipids, regulation of oxygen delivery through 2,3-diphosphoglycerate (2,3-DPG) in red blood cells, and buffering of acid-base balance [1,2]. Severe hypophosphatemia, defined as serum phosphate levels below 0.32 mmol/L, can result in life-threatening complications, including impaired diaphragmatic contractility, respiratory muscle weakness, arrhythmias, hemolysis, rhabdomyolysis, altered mental status, seizures, and even death if left untreated [3-5]. The prevalence of hypophosphatemia varies depending on the population studied, ranging from 2% in general hospitalized patients to as high as 30-60% in critically ill patients admitted to intensive care units [6,7]. Despite this, it is often underdiagnosed due to nonspecific symptoms and the lack of routine phosphate measurement in acute presentations unless specifically suspected. Hypophosphatemia can occur due to three primary mechanisms: reduced intestinal absorption, increased renal excretion, or intracellular redistribution [8]. Classical causes include malnutrition, alcohol misuse, vitamin D deficiency, renal tubular disorders, and refeeding syndrome, while redistribution secondary to hyperventilation-induced respiratory alkalosis remains an underrecognized cause. During hyperventilation, excessive CO_2_ loss leads to respiratory alkalosis, which stimulates phosphofructokinase (PFK), a rate-limiting enzyme in glycolysis, thereby accelerating glucose metabolism and driving phosphate into cells, markedly lowering extracellular phosphate concentrations despite normal total body stores [9,10]. Anxiety-induced hyperventilation is a frequent presentation in emergency departments and is often misattributed solely to psychiatric causes; however, its metabolic consequences, including severe hypophosphatemia and mild lactate elevation during respiratory alkalosis, are rarely considered [11]. The overlap between panic symptoms, such as dyspnea, paresthesia, dizziness, and palpitations, and underlying biochemical disturbances further complicates recognition. We present the case of a 38-year-old male patient with a background of anxiety disorder and Crohn’s disease who developed severe hypophosphatemia secondary to hyperventilation during an acute panic episode, underscoring the importance of considering metabolic disturbances in anxiety-related presentations and the need for prompt recognition, investigation, and targeted management to prevent potentially life-threatening complications [12].

## Case presentation

A 38-year-old male patient was referred to the Accident and Emergency (A&E) department by his general practitioner (GP) following abnormal blood test results that revealed severe hypophosphatemia (serum phosphate <0.20 mmol/L; reference: 0.8-1.5 mmol/L) and a mildly elevated lactate level (2.4 mmol/L; reference: 0.5-2.2 mmol/L). He reported recurrent episodes of shortness of breath, hyperventilation, tingling sensations in his extremities, and near-syncope, all of which had worsened over recent weeks. He also described frequent sugar cravings, intermittent diarrhea with six to eight loose stools per month, and increasing anxiety at the time of presentation, consistent with his psychiatric history.

The patient’s medical history was significant for Crohn’s disease, which had been in remission, and generalized anxiety disorder, for which he was prescribed citalopram 20 mg daily. He reported recent weight gain of approximately 6 kg over the past three months and intermittent muscle cramps over the last year. There was no history of alcohol misuse, illicit drug use, excessive antacid intake, or recent dietary changes, all of which are recognized contributors to electrolyte disturbances [1,2].

**Figure 1:**
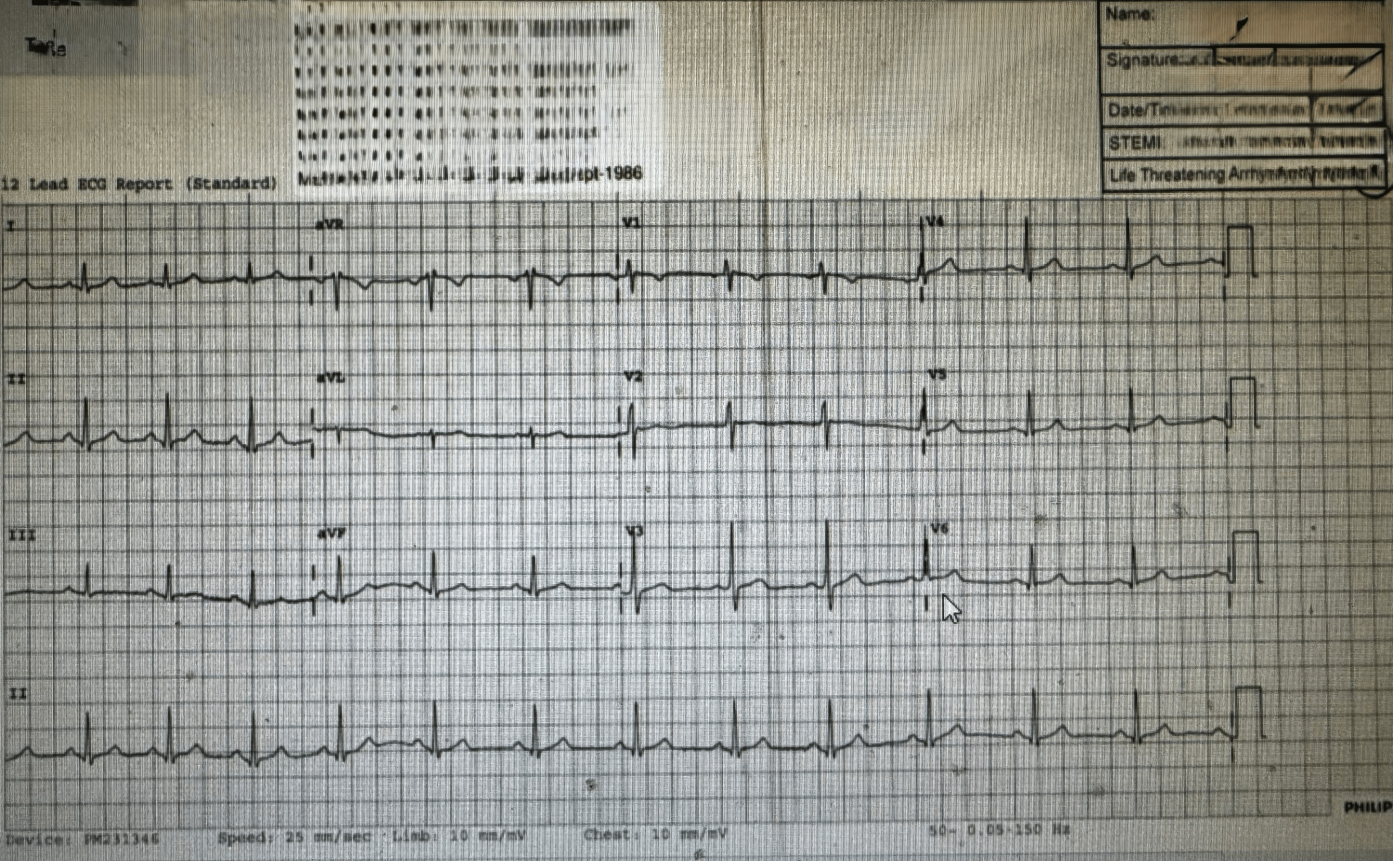
Electrocardiogram (ECG) at presentation The 12-lead ECG demonstrates sinus tachycardia at a rate of approximately 108 beats per minute, with normal P-wave morphology, narrow QRS complexes, and no acute ST-segment or T-wave abnormalities. There is no evidence of ischemia, arrhythmia, or conduction block. These findings are consistent with the patient’s hyperventilation and anxiety state rather than a primary cardiac pathology.

On examination, the patient appeared visibly anxious and tachypneic but was alert and oriented. His vital signs showed sinus tachycardia at 108 bpm (reference: 60-100 bpm), blood pressure of 128/78 mmHg (reference: 90/60 to 140/90 mmHg), a respiratory rate of 28 breaths per minute (reference: 12-20 breaths/min), oxygen saturation of 98% (reference: ≥95%) on room air, and a temperature of 36.7°C (reference: 36.1-37.2°C). Neurological examination revealed mild perioral numbness and carpopedal spasms, features commonly associated with acute electrolyte imbalances [3]. Abdominal examination was unremarkable, with no tenderness or organomegaly. His ECG showed a normal sinus rhythm (Figure 1).

Laboratory investigations demonstrated a critically low serum phosphate level <0.20 mmol/L (reference: 0.8-1.5 mmol/L) and elevated lactate 2.4 mmol/L (reference: 0.5-2.0 mmol/L). Arterial blood gas analysis revealed a pH of 7.535 (reference: 7.35-7.45), consistent with respiratory alkalosis. Serum calcium was mildly elevated at 2.6 mmol/L (reference: 2.1-2.6 mmol/L), while magnesium, renal, and liver function tests were all within normal ranges. Thyroid function testing revealed a normal thyroid-stimulating hormone (TSH) level of 1.5 mU/L (reference: 0.4-4.0 mU/L). Table 1 shows the investigations.

**Table 1:** Lab investigations TSH: Thyroid-stimulating hormone; ALT: Alanine transaminase; AST: Aspartate aminotransferase. The table summarizes the initial biochemical and arterial blood gas findings, with reference ranges for context. The patient demonstrated critical hypophosphatemia, mild hypercalcemia, respiratory alkalosis, and elevated lactate levels—findings that guided the clinical diagnosis and management.

Investigation	Patient's value	Reference range	Interpretation
Serum phosphate	<0.20 mmol/L	0.8-1.5 mmol/L	Critical hypophosphatemia
Serum lactate	2.4 mmol/L	0.5-2.0 mmol/L	Mild elevation
Arterial pH	7.535	7.35-7.45	Respiratory alkalosis
Serum calcium	2.6 mmol/L	2.1-2.6 mmol/L	Upper limit of normal
Serum magnesium	1 mmol/L	0.7-1.0 mmol/L	Within normal limits
TSH	1.5 mU/L	0.4-4.0 mU/L	Normal thyroid function
Renal function tests	Normal	Creatinine: 60-110 µmol/L	Within normal limits
Liver function tests	Normal	ALT: <40 U/L; AST: <40 U/L	Within normal limits

Given the clinical picture, several differential diagnoses were considered, including malabsorption syndromes such as a Crohn’s disease flare or celiac disease, excessive gastrointestinal phosphate loss from vomiting or diarrhea, hyperparathyroidism, Fanconi syndrome, and refeeding syndrome [4-6]. However, there was no biochemical or clinical evidence of renal phosphate wasting, endocrine dysfunction, or impaired intestinal absorption.

The final diagnosis was severe hypophosphatemia secondary to hyperventilation-induced respiratory alkalosis. The biochemical disturbance was attributed to an anxiety-related hyperventilation episode, which caused excessive CO_2_ loss, a rise in blood pH, and stimulation of glycolysis, leading to an intracellular shift of phosphate [7-9].

The patient was admitted for urgent management. Intravenous phosphate replacement was initiated, with close serial monitoring of electrolytes to avoid complications such as hypocalcemia and metastatic calcification [10]. Within 24 hours, serum phosphate levels began to improve, and the patient’s neuromuscular symptoms subsided as anxiety was controlled with reassurance and breathing techniques. Once stabilized, he was transitioned to oral phosphate supplementation for maintenance and discharged with outpatient follow-up. At review, biochemical parameters had normalized, and the patient remained clinically well.

## Discussion

Hypophosphatemia is a relatively uncommon but clinically significant biochemical disturbance encountered in acute medicine. Serum phosphate plays a crucial role in cellular energy production, membrane integrity, and acid-base homeostasis. Its deficiency can result from decreased intestinal absorption, increased renal losses, or redistribution into the intracellular compartment [1]. In our patient, the cause of severe hypophosphatemia (<0.20 mmol/L) was linked to hyperventilation-induced respiratory alkalosis secondary to an acute anxiety episode, resulting in a profound intracellular shift of phosphate.

Pathophysiology of hyperventilation-induced hypophosphatemia

Hyperventilation leads to excessive elimination of CO_2_ through the lungs, reducing the arterial partial pressure of CO_2_ (PaCO_2_) and resulting in respiratory alkalosis [2]. The fall in PaCO_2_ shifts the bicarbonate buffer equilibrium - CO_2_ + H_2_O ⇌ H_2_CO_3_ ⇌ H^+^ + HCO_3_^-^ - toward the left, thereby decreasing the hydrogen ion concentration and increasing blood pH.

This alkalotic state triggers several intracellular metabolic adaptations. Alkalosis activates PFK, a rate-limiting enzyme in glycolysis, which accelerates glucose metabolism and increases intracellular phosphate demand [3]. Phosphate is a key substrate for the synthesis of ATP and 2,3-DPG, both essential for cellular energy metabolism and oxygen release from hemoglobin. As glycolytic activity rises, phosphate shifts from the extracellular space into cells, leading to severe hypophosphatemia despite normal total body phosphate stores [1,4].

**Figure 2:**
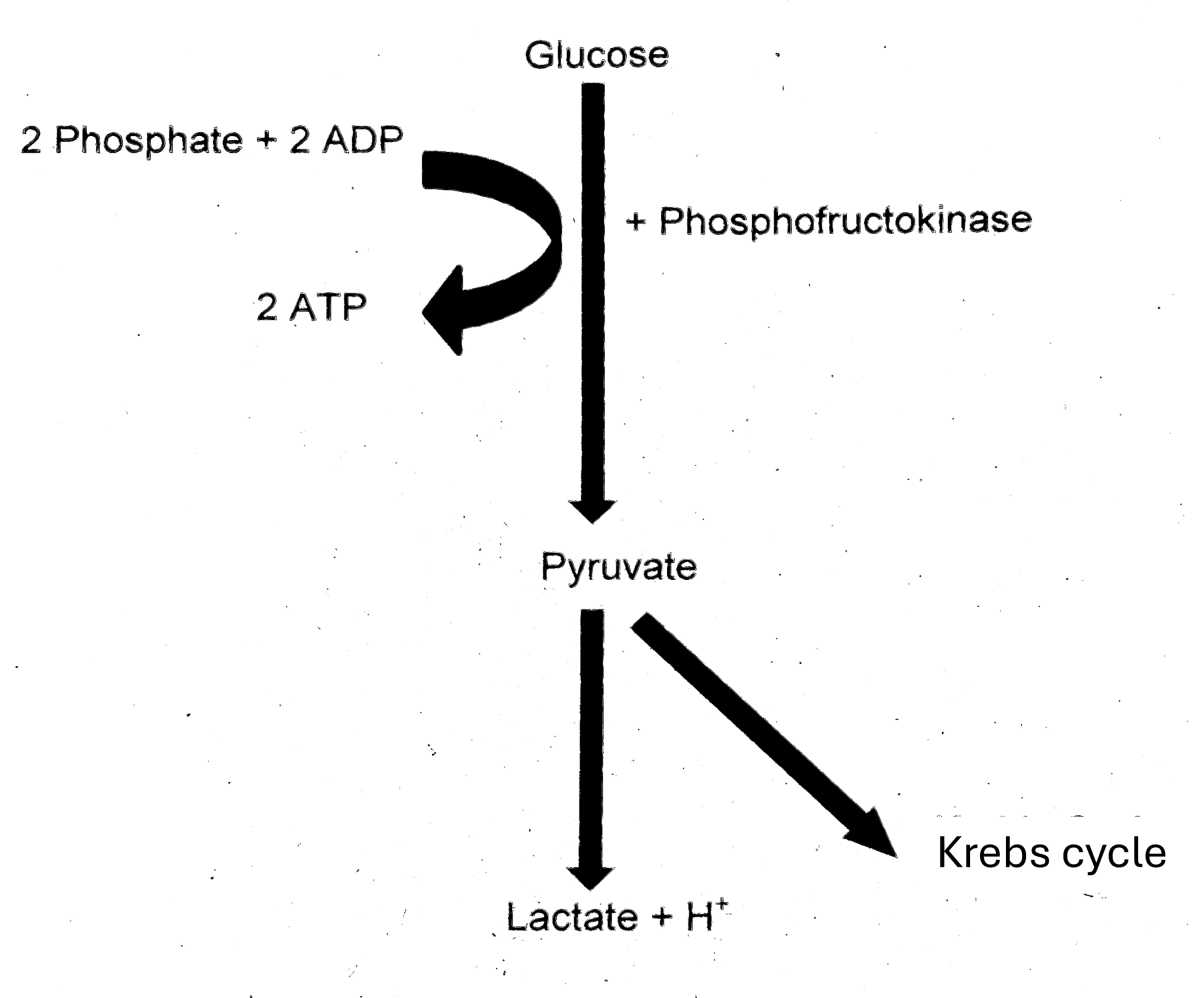
Glycolytic pathway Schematic representation of the glycolysis pathway. Glucose undergoes phosphorylation and sequential enzymatic reactions (including phosphofructokinase activity) to form pyruvate. Pyruvate then enters either anaerobic metabolism, yielding lactate and hydrogen ions, or aerobic metabolism via the Krebs cycle. The process consumes inorganic phosphate and adenosine diphosphate (ADP), generating adenosine triphosphate (ATP), thereby linking phosphate availability to cellular energy production. Image credit: Created by the authors using Microsoft Word (Microsoft Corp., Redmond, WA, US).

Furthermore, the increased glycolytic flux enhances the conversion of pyruvate to lactate under relative mitochondrial constraint, explaining the mild lactate elevation (2.4 mmol/L) observed in this patient. The sequence of these metabolic events is illustrated in Figure 2, showing the role of PFK in driving glycolysis and phosphate consumption.

**Figure 3:**
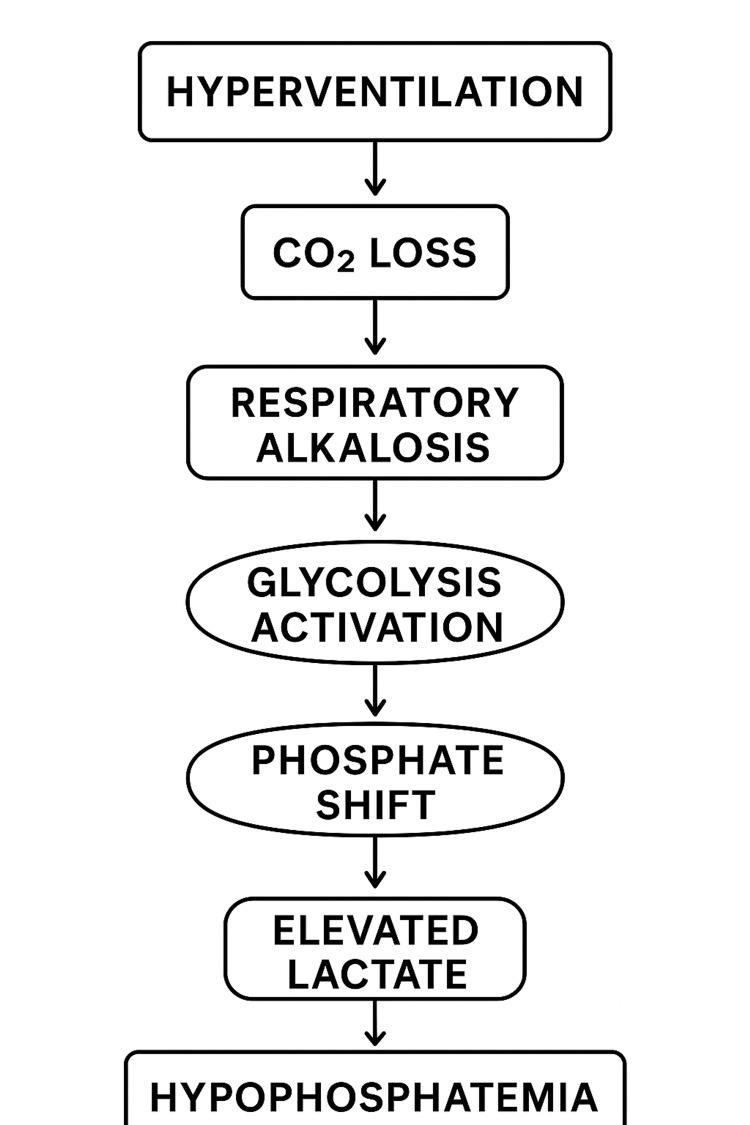
Pathophysiological cycle of hyperventilation-induced hypophosphatemia Hyperventilation leads to CO_2_ loss and respiratory alkalosis, which activates glycolysis and drives phosphate into cells, resulting in severe hypophosphatemia. Image credit: Created by the authors using Microsoft Word (Microsoft Corp., Redmond, WA, US).

Figure 3 shows the pathophysiological cycle that leads to hypophosphatemia and elevated lactate. 

This pathophysiological mechanism has been described in several clinical studies. O’Brien and Coberly [4] reported a similar presentation of severe hypophosphatemia in respiratory alkalosis, in which hyperventilation alone caused a profound intracellular phosphate shift without evidence of malabsorption, renal loss, or hormonal abnormalities. Maddock [5] also described the lactic acid response to alkalosis in patients with panic disorder, demonstrating that panic-related hyperventilation can reproduce these metabolic derangements. In our patient, hyperventilation during an acute anxiety episode likely initiated the same cascade, culminating in severe hypophosphatemia (<0.20 mmol/L).

Clinical implications of severe hypophosphatemia

**Figure 4:**
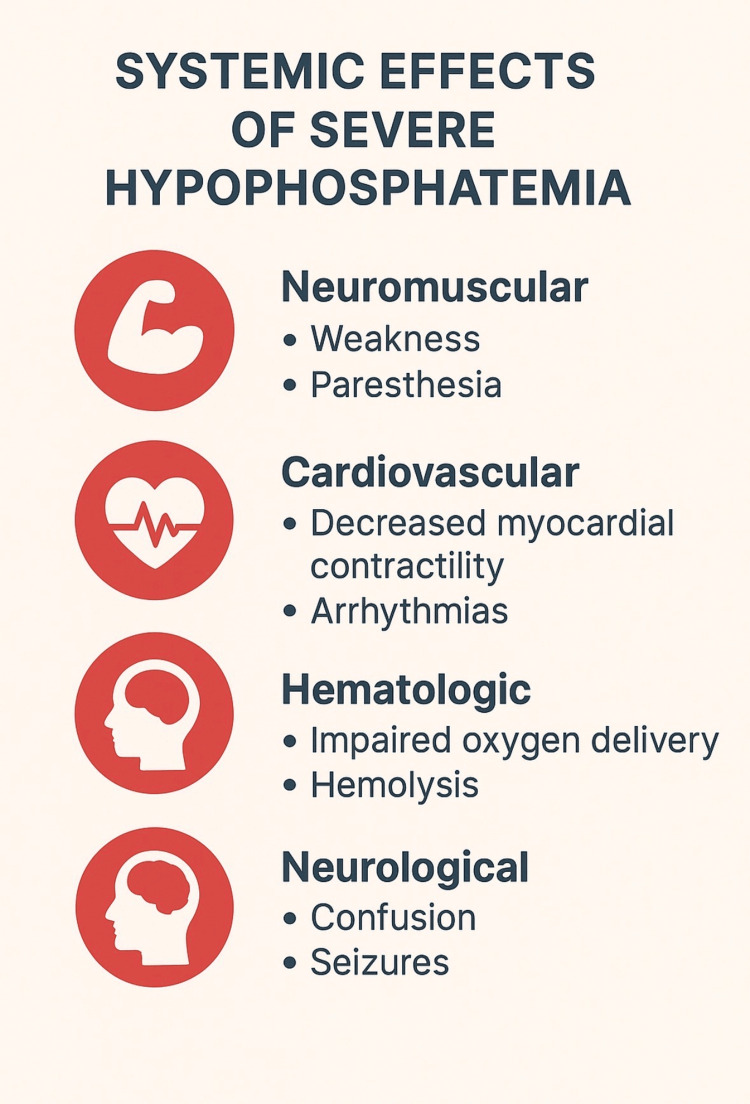
Systemic effects of severe hypophosphatemia Neuromuscular, cardiovascular, hematologic, and neurological complications are illustrated. Image credit: Created by the authors using Microsoft Word (Microsoft Corp., Redmond, WA, US).

Severe hypophosphatemia has important consequences for multiple organ systems because phosphate is central to energy metabolism, cellular signaling, and oxygen delivery. When serum phosphate levels fall below 0.4 mmol/L, the effects can be clinically significant and sometimes life-threatening (Figure 4).

From a neuromuscular perspective, patients may develop generalized weakness, fatigue, and paresthesia. In more severe cases, depletion of phosphate impairs diaphragmatic contractility and skeletal muscle function, leading to respiratory muscle fatigue and, potentially, respiratory failure. These symptoms were mirrored in our patient, who experienced breathlessness, carpopedal spasms, and tingling sensations, reflecting the impact of acute phosphate depletion on neuromuscular excitability.

Cardiovascular complications are also well-documented. Phosphate deficiency reduces myocardial contractility and increases the risk of arrhythmias due to impaired ATP availability within cardiac tissue. Although our patient’s ECG revealed sinus tachycardia without arrhythmic changes, the literature describes cases of hypophosphatemia precipitating serious rhythm disturbances and reduced cardiac output.

Hematologic consequences occur because phosphate is required for the synthesis of in the red blood cells. Reduced 2,3-DPG shifts the oxygen-hemoglobin dissociation curve to the left, resulting in impaired oxygen delivery to tissues. In addition, severe hypophosphatemia may cause hemolysis due to instability of erythrocyte membranes.

Neurologically, patients may develop confusion, irritability, altered mental status, and in extreme cases, seizures or coma. These complications highlight how systemic and wide-reaching the impact of phosphate depletion can be, even in the absence of underlying structural disease.

In the context of our case, the patient’s symptoms of near-syncope, anxiety, and neuromuscular irritability were best explained by these acute systemic effects of hypophosphatemia. This underscores the importance of early recognition and correction of phosphate disturbances in patients presenting with hyperventilation or panic-related episodes to prevent progression to more severe complications [8].

Anxiety, panic, and hyperventilation

Hyperventilation is frequently associated with panic attacks and anxiety disorders. The Diagnostic and Statistical Manual of Mental Disorders (DSM-IV) describes panic attacks as episodes of intense fear associated with somatic symptoms such as dyspnea, dizziness, and paresthesia [9]. In such cases, hyperventilation is both a symptom and a driver of biochemical disturbances.

Reynolds et al. [10] highlighted that hyperventilation can induce metabolic alkalosis severe enough to produce electrolyte imbalances, particularly hypophosphatemia and hypocalcemia. Our patient similarly exhibited panic-induced hyperventilation, leading to significant derangement in phosphate metabolism.

Management considerations

The management of severe hypophosphatemia involves a two-pronged approach: correction of the underlying cause and restoration of phosphate balance. In this patient, intravenous phosphate replacement was promptly initiated due to the critically low serum phosphate (<0.20 mmol/L) and neuromuscular symptoms [6]. Electrolyte levels were closely monitored to prevent complications such as hypocalcemia, soft-tissue calcification, and cardiac arrhythmias, which may occur with rapid phosphate repletion [1,7].

Once serum phosphate improved, the patient was transitioned to oral phosphate supplementation for maintenance therapy and gradual replenishment of intracellular stores. Simultaneously, management of the underlying anxiety and hyperventilation was prioritized. The patient benefited from guided breathing techniques, reassurance, and continuation of citalopram, which stabilized anxiety symptoms and reduced further hyperventilation episodes.

Previous studies emphasize the importance of early recognition of hypophosphatemia in patients presenting with panic or anxiety-induced hyperventilation. Maddock [5] and Geerse et al. [6] reported that unrecognized phosphate depletion can result in serious complications, including rhabdomyolysis, cardiac dysfunction, and respiratory failure, particularly in acutely ill patients. Therefore, routine electrolyte assessment is crucial in such cases to ensure timely intervention.

**Figure 5:**
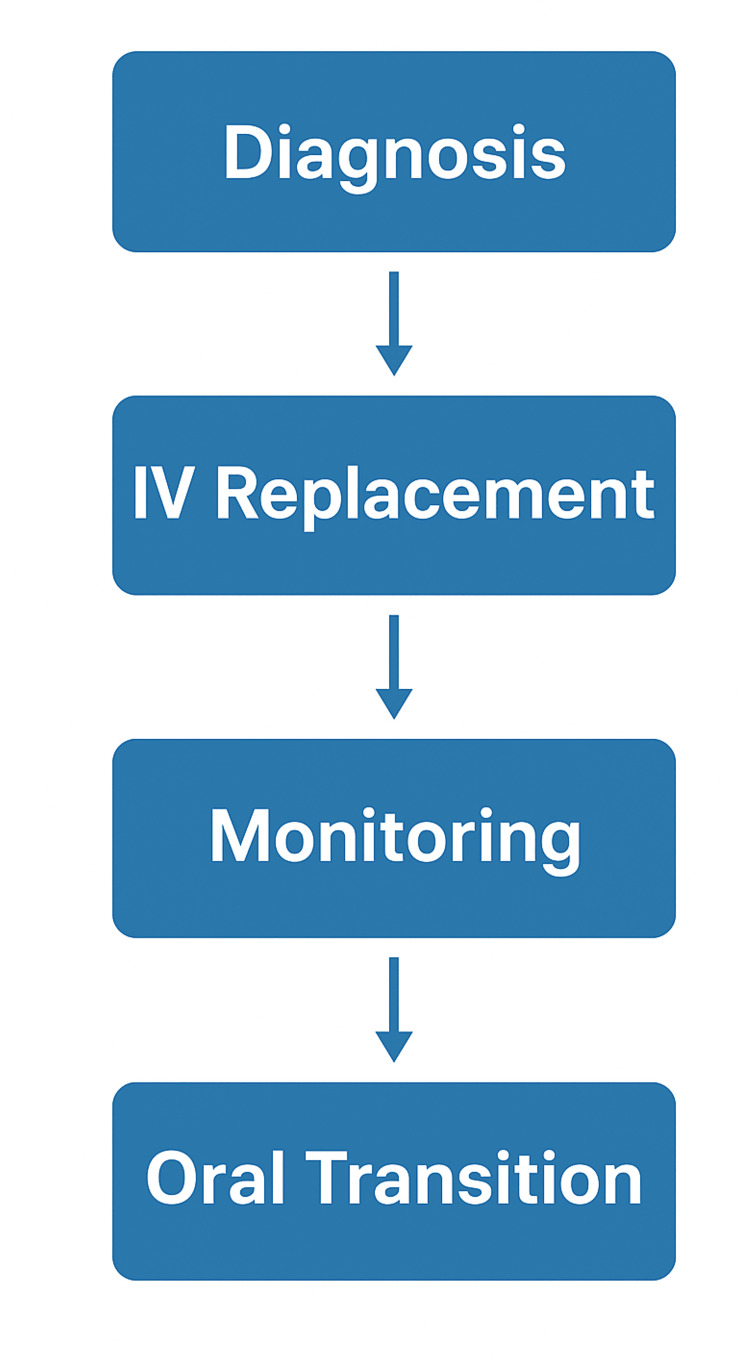
Management flowchart for severe hypophosphatemia The diagram illustrates key steps in the clinical management of severe hypophosphatemia, including diagnosis, intravenous phosphate replacement, serial monitoring, transition to oral supplementation, and recovery. Image credit: Created by the authors using Microsoft Word (Microsoft Corp., Redmond, WA, US).

The stepwise approach to diagnosis and management in this case is summarized in Figure 5, which outlines the clinical workflow from presentation to full biochemical and symptomatic recovery.

Comparison with existing literature

While most cases of hypophosphatemia occur due to malnutrition, alcohol misuse, or renal losses, hyperventilation-induced hypophosphatemia remains underrecognized in acute care [6,13]. Our findings are consistent with Kligler et al. [14], who reported that panic-induced hyperventilation can produce significant metabolic shifts, even in patients without underlying psychiatric disease. Increased awareness among acute care clinicians is essential to ensure prompt diagnosis and treatment.

Clinical relevance

This case highlights the importance of considering metabolic causes in patients presenting with panic-like symptoms. Routine assessment of electrolytes, especially phosphate, in patients with hyperventilation can prevent potentially life-threatening complications.

## Conclusions

This case highlights hypophosphatemia as a potential yet underrecognized complication of hyperventilation in acute anxiety. The phosphate depletion resulted from an intracellular shift due to respiratory alkalosis rather than nutritional or renal causes. It emphasizes that anxiety-induced hyperventilation can cause serious biochemical disturbances, warranting prompt recognition and management to prevent complications such as weakness, arrhythmias, and neurological symptoms.

Clinicians should therefore assess electrolytes in acute anxiety presentations and not attribute symptoms solely to psychological causes. The rapid recovery following phosphate correction underscores the importance of early intervention and coordinated care between emergency, internal medicine, and psychiatry teams. Routine consideration of phosphate levels in severe hyperventilation may help avert preventable morbidity.
